# Speculative Expropriation

**DOI:** 10.1177/00905917251398813

**Published:** 2026-01-26

**Authors:** Wayne Wapeemukwa

**Affiliations:** 1 The University of British Columbia, Vancouver, BC, Canada

**Keywords:** Speculative expropriation, settler colonialism, primitive accumulation, original accumulation, ground rent, Indigenous dispossession, Marx

## Abstract

This article introduces the concept of “speculative expropriation” to reframe Marx’s analysis of expropriation in the context of settler colonialism and capitalism in the West. I begin by examining Marx’s ideas of primitive accumulation and original expropriation, showing how his incomplete analysis of social relations to land can be extended to the trans-Atlantic context. Drawing on Marx’s pre-*Capital* manuscripts, I examine his treatment of ground rent and its relationship to feudalism, highlighting how the development of capitalism in the West relied on mechanisms overlooked in conventional readings of Marx’s work. I argue that settler colonialism in the New World did not follow the same logic as European primitive accumulation, where land was taken from peasants. Instead, speculative expropriators gave land to settlers, but only under specific racial and gendered conditions that tied landowners to the financial and political systems of the settler state. The land was often granted with mortgages and preemptive land sales, which placed settlers in debt and subjected them to the control of colonial institutions. This financial framework, grounded in the dispossession and genocide of Indigenous peoples, allowed capitalism to expand through speculative real estate and colonial constructs. By theorizing speculative expropriation, I show how the dynamics of settler colonialism reinvented capitalist accumulation, turning land ownership into a means of controlling settlers through debt rather than directly extracting their labor. This framework provides a new lens for understanding how capitalism expanded across the Atlantic and solidified its global reach in tandem with Indigenous dispossession and population replacement.

## Introduction

But it isn’t the situation in the colonies that concerns us here. All we are interested in is a secret that the political economy of the Old World discovered in the New World and loudly proclaimed. The capitalist mode of production and accumulation, and thus also capitalist private property, requires that private property based on a person’s own labor be destroyed–in other words, that the workers be expropriated.^1^

The North American “West” surfaces at the horizon of Marx’s *Capital*, Vol. I, in this terminal sentence. There, Marx concludes that capitalism escapes the “Old World,” but does not explain how it interfaces with settler colonialism in the “New World,” or how capitalist relations of property and dependency are installed despite land’s abundance. While the West serves Marx as a vivid example of “Primitive Accumulation,” he elsewhere claims that its context of broadcast ownership countervails his analysis of ground rent, and may even ring the “death-knell” of Europe’s aristocracy, since land has not been separated from labor, and landed property does not confront the worker as an “alien power and a barrier.”^2^ Thus, the West figures cacophonously as both confirmation and contradiction: a frontier that is both central, yet peripheral, to Marx’s critique of the capitalist mode of production, as well as its historical presupposition, landed property.

Marx’s writings on capitalist relations to land are incomplete and incongruous. In his *Manuscripts of 1864-65*–his only draft of *Capital*, Vol. III–he claims that “primitive accumulation [and] the monopoly of landed property is a *historical precondition* for the capitalist mode of production and remains its permanent *foundation*,” then tantalizingly adds: “But the form of *landed property* which greets the capitalist mode of production at the start does not correspond to this mode.”^3^ Marx analyzes capitalism’s “foundation” in “Primitive” or “Original Accumulation,” a historical-epilogue concluding *Capital*, Vol. I, breaking from its prior theoretical schema of production and labor power exploitation. There, Marx vividly chronicles capitalism’s violent genesis but does not illustrate how “feudal landed property [is] transformed into the *economic* form corresponding to this mode of production.”^4^ Indeed, this is the purpose behind his subsequent, and misunderstood, analysis of ground rent.

Marx disseminates his analysis of landed property and ground rent across voluminous notebooks and protean drafts, including *Grundrisse*, the *Manuscripts of 1861-63*, the *Manuscripts of 1864-65*, and *Capital*, Vol. III, which Engels editorialized in 1894–nearly 30 years after Marx wrote the original!^5^ Evidently, the expropriation of the West resulted in a New World of property, but the journey to that world took detours that defy schematic predictions about the trans-historical succession of modes of production, as well as capitalism’s normative logic of accumulation. The problem is that these detours, scattered across manuscripts and long obscured by Engels’s editorial interventions–not to mention Marx’s heavily mathematized presentations–have rarely been synthesized into the coherent theory they were intended to be. As a result, Marx’s crucial insight into how capitalism interfaces with noncapitalist modes and non-European social formations remains ignored. This study fills that gap by theorizing the concept of *speculative expropriation*.

This article begins with the intuition that Marx’s incomplete analysis of social relations to land still offers a framework for rethinking the articulation of settler colonialism with capitalism in the West. I first contextualize Marx’s writings on expropriation with what has been referred to as “Primitive Accumulation,” then disclose Marx’s self-selected alternative: “Original Expropriation.” Next, I reconstruct Marx’s analysis of ground rent from his pre-*Capital* manuscripts–sources that have been neglected in favor of the editorialized analysis provided in *Capital*, Vol. III–to show how capitalism interfaces with noncapitalist modes, specifically feudalism. Finally, I concur with Marx that his analyses of original accumulation and ground rent are limited to Western Europe and that a theory of expropriation particular to the trans-Atlantic West is necessary. Therefore, I offer a generative mechanism that captures how capitalism overspills the Atlantic via speculative-financial constructs I name: “speculative expropriation.”

With speculative expropriation, I theorize how settler colonialism in the West reinvented capitalism, turning abundance into dependence, not by *taking* the land away, as it did in Europe, but by *giving* it away. Throughout his critique of capital, Marx insists that expropriating direct agricultural producers is central to capitalist development and that expropriators never let land go without compensation. In contrast, Old World primitive accumulationists extracted land from peasants, while New World speculative expropriators distributed it–sometimes nearly for free–after dispossessing and incarcerating Indigenous proprietors. Instead of barring settlers from the land, they actively sought to *emplace* them, but only under specific racial and gendered conditions. Eligible proprietors held land thanks to financial constructs, such as the mortgage, which tethered them to the infrastructures and institutions of the neophyte settler state. In this New World–made possible only by Indigenous genocide–debt and credit sufficed to control and exploit an obstreperous class of direct producers. But this speculative expropriation did not end with the Western, “American” frontier; it continues to underwrite dispossession presently and globally.

## Original Expropriation

Beginning in the mid-1840s, and especially after his 1845 trip to see Engels in Manchester, Marx conceives capitalism as a system of class-based expropriation. Although the literal term “expropriation” does not figure explicitly in the *Economic and Philosophic Manuscripts of 1844*, the sensualist concept of “estranged labor” follows his excursus on ground rent and concludes the first manuscript. In 1847–48, in *The Poverty of Philosophy* and *The Communist Manifesto*, he conceives expropriation in terms of a class war between capitalists and workers, identifying that war’s *casus belli* with an original expropriation of the land. But the most succinct formulation of Marx’s early idea of expropriation lies in *The German Ideology*, where he stakes a claim on a materialist-teleological conception of history that “appears to be contradicted by the fact of conquest.”^6^ But he overturns this thesis in *Grundrisse* and, in *Capital*, Vol. I, writes definitively: “The separation of property and labor becomes the necessary consequence of a law that seemed to proceed from the identity of those things.”^7^ Ultimately, Marx reconceives expropriation not merely as a moral injustice but as the locomotive of class domination and social transformation, even as it stands in tension with accumulation.

### Original Expropriation

Marx insists on expropriation in a two-part lecture to the International in June 1865: *Value, Price, and Profit*. Nearly lost until discovered by his daughter and son-in-law in 1898, this précis condenses his entire critique of political economy.^8^ Delivered in English, it covers all his central concepts: the labor theory of value, class struggle, and, most importantly, “primitive accumulation.”

In the chapter on “Labor Power,” Marx argues that while every commodity can be evaluated by labor power, labor power itself cannot. Since labor creates and measures value, attempts to determine the value of labor are tautological; they presuppose a concept of value independent of labor. Rather than succumbing to this circularity, Marx—much as in his critique of Proudhon—rises above the circularity to posit a theory of how this self-positing misrepresentation must have arisen in the first place:

How does this strange phenomenon arise, that we find on the market a set of buyers, possessed of land, machinery, raw material, and the means of subsistence [. . .] and on the other hand, a set of sellers who have nothing to sell except their labouring power, their working arms and brains? [. . .] The inquiry into this question would be an inquiry into what the economists call “previous or original accumulation,” but which ought to be called *original expropriation*. We should find that this so-called original accumulation means nothing but a series of historical processes, resulting in a decomposition of the original union existing between the labouring Man and his Instruments of Labour [. . .]. The separation between the Man of Labour and the Instruments of Labour once established, such a state of things will maintain itself and reproduce itself upon a constantly increasing scale, until a new and fundamental revolution in the mode of production should again overturn it, and restore the original union in a new historical form.^9^

Marx explicitly refers to “original expropriation.” Writing in English, he intercepts translational debates and deploys ecological metaphors, stressing how capital “decomposes” precapitalist relations to the land. Although capitalist relations dominate, he insists they do not erase precapitalism. Instead, communism must redirect capitalism’s productive forces toward “a new and fundamental revolution” whose objective is to “restore the original union in a new historical form”—not a regression to a bygone past, but the rehabilitation of precapitalist social relations to invent something new. This is no nostalgic restoration, but a creative rearticulation of extant and imperiled social relations into a “new historical form.” Marx envisions neither a return to a mythical past nor a stadial march into the industrial future but a conscious reconstitution of social relations to the land despite their “decomposition.”^10^ Yet if Marx could glimpse in noncapitalist relations the seeds of a new historical form, he also knew that political economy cloaked their violent destruction under the myth of “original accumulation.”

### Original Accumulation

Marx’s recognition that noncapitalist social relations endure helps explain his critique in *Capital*, Vol. I, of the myth of “original” or “primitive” accumulation.

But what appears as a mythical beginning is in fact an ongoing and, indeed, existential problem. Lenin and Luxemburg cast the period of original accumulation as a stadial meat-mincer through which all societies must pass–a misunderstanding recently criticized by David Graeber and David Wengrow.^11^ David Harvey redefines it as “accumulation by dispossession,” an ongoing feature of capitalism, but in positing perpetual dependence on an external “outside,” he repeats Luxemburg’s reductionist misstep.^12^ Massimiliano Tomba, by contrast, emphasizes that treating original accumulation as a singular, diachronic origin misses Marx’s point: capitalist time synchronizes precapitalist and capitalist forms across history, so “original” accumulation is not simply a beginning but an *ongoing* temporal relation.^13^ Jason Read and Kevin B. Anderson stress that Marx limited his analysis to Western Europe,^14^ while Amy Allen argues that he remained committed to a multilinear yet Eurocentric vision in which capitalism appears the most developed path,^15^ But the stakes were already very clear in Marx’s suppressed correspondence with Vera Zasulich: must extant, noncapitalist social relations to land submit to capitalist violence before socialism, or could such social relations bypass original/primitive accumulation altogether?

Recent translations of Marx’s *Capital*, Vol. I, and post-1868 notebooks have reanimated this vital debate. For instance, in her correspondence with Marx, Zasulich cites the 1867 first edition of *Capital*, Vol. I, which was the basis for the 1872 Russian translation. But, Anderson observes that Marx cites the 1872–1875 French edition of *Le Capital* in his response to Zasulich and singles out a crucial paragraph that Marx continued revising for international audiences.^16^ Zasulich’s paragraph from the German first edition/Russian translation reads:

*Die Expropriation der Arbeiter von Grund und Boden bildet die Grundlage des ganzen Prozesses. Wir haben sie also zuerst zu betrachten. Ihre Geschichte nimmt in verschiedenen Phasen in verschiedner Reihenfolge. Nur in England, das wir daher also Beispiel nehmen, besitzt sie klassiche Form.* ^17,18^

This passage is reproduced verbatim in the 1872 second edition, which also serves as the basis for the new 2024 English translation by Paul North and Paul Reitter, except for two minor changes with significant consequences. First, Marx replaces “Arbeiter von Grund und Boden [workers from land and soil]” with “ländlichen Produzenten [agricultural producers],” a change he retains and translates into French as “*cultivateurs* [farmers].” This change signifies that Marx is thinking in more capacious terms than an industrial proletariat. Secondly, Marx adds four words after qualifying that original accumulation happens in “*verschiedenen Phasen in verschiedner Reihenfolge* [different phases and different countries],” adding “*und in vershiedenen Geschichtsepochen* [and in different historical epochs].”^19^ In *Le Capital*, Marx retains this qualification as, “*ou suivre un ordre de succession different* [or follows a different order of succession].” With these small additions, Marx emphasizes that capitalist development varies not just by country or phase but can even occur at different historical times, and in hybrid forms shaped by particular and unique histories and contexts. Those, like Anderson and Allen, who debate whether Marx is a *uni*-linear or *multi*-linear thinker miss a more fundamental shift in his thinking: For Marx, there is *no* single timeline every society must follow since capitalist development is *non*linear.

Because of these changes, contemporary readers of Marx, such as Anderson and William Claire Roberts,^20^ claim that *Le Capital* is preferable to *Das Kapital*. However, Marx also makes consequential additions to the French that are not included in the German and, thus, the recent English translation by North and Reitter. In the German “Historical Tendency of Capitalist Accumulation,” Marx writes: “In the realms of both production and society, this mode of industry is compatible only with narrow, spontaneously arising limits. Once it reaches a certain level, it brings into being the material means of its own destruction.”^21^ Yet, in *Le Capital*, Marx plays to his audience and adds a quote from the French political economist Constantin Pecqueur: “*L’éterniser, ce serait, comme le dit pertinemment Pecqueur, ‘décréter la médiocrité en tout* [To eternalize it would be, as Pecqueur aptly says, ‘to decree mediocrity in everything].’”^22^ Although some identify *Le Capital* as more capacious than *Das Kapital*, here Marx seems to be evidencing the opposite.

Nevertheless, Marx argues that expropriation leads to uneven results. He explains how landlords invented laws to dispossess peasants and galvanize them into a historically unique proletariat. However, these peasants were not proletarianized automatically; in fact, many remained dispossessed, without access to traditional means of reproduction. Expropriating direct agricultural producers resulted in *two* historically unique classes, therefore: a free and rightless working class defined by new relations of capitalist production *and* another class of nonworkers defined by the total lack of relations to traditional reproduction–a class whom Fanon later christens “the wretched.” As Peter Hudis has aptly surmised, proletarianization presupposes expropriation, but the reverse is not necessarily true.^23^ So the mode of production Marx elucidates in *Capital* is a description of one corner of the world–an important corner, certainly, but not a template. Marx sees no necessary correspondence between feudalism and capitalism, yet this does not mean he commits to non-correspondence, either. For isn’t it the case that industrialized societies, such as England, expropriate more ruthlessly? Doesn’t the very concentration of capital intensify expropriation rather than diminish it?

What is still needed is a non-teleological theory of expropriation that avoids economic determinism as well as political realism.

## Ground Rent

As is well known, Marx provides a theory of rent in the lengthy penultimate section of *Capital*, Vol. III. But that presentation is misleadingly self-contained since Engels editorialized it more than thirty years after Marx wrote the original in 1865, which was based on an even prior analysis from 1862. Engels even posthumously exegeted the forty-third chapter from Marx’s scattered, disorganized notes. In fact, Marx’s study of rent not only overspills *Capital*, Vol. III, but can be traced back to 1843, with discussions appearing in the first and second manuscripts of 1844. In 1857, Marx reexamined rent and even planned to dedicate the second of his six-volume *Contribution* to an investigation into landed property but was thrown off track by chance.

Serendipitously, on June 9, 1862, Ferdinand Lassalle sent a letter to Marx requesting that he return a loaned book by Karl Johann Rodbertus.^24^ When Lassalle’s request was delivered, Marx was in the middle of studying the concept of relative surplus value but had to pause and digress into Rodbertus so as to return his comrade’s book in good order. Of course, as was typical for him, Marx totally failed to fulfill Lassalle’s request and instead embarked on a year-long serpentine investigation into rent that not only formed the core of his analysis in volume three but also what was packaged into and retroactively labelled *Capital*, Vol. IV: *Theories of Surplus Value*. Although Marx calls James Anderson “the true originator of the modern theory of rent,”^25^ his “accidental” and “unexpected” digression into Rodbertus finally equipped him with the tools necessary to explain capitalized rent.^26^ In his authoritative reading of Marx’s *Manuscripts of 1861-63*, Enrique Dussel refers to this random digression as the “central moment.”^27^

### Anatomy of the Ape

In his *Economic and Philosophic Manuscripts of 1844*, Marx posited that landed property and capital originate from distinct modes of production that interface with bourgeois society but left their interstices unanalyzed. In *Grundrisse*, he returns to this question and outlines a method of analysis that follows the *inner* logic of capital’s relationship to landed property, which is not necessarily the chronological order in which they appear in history. So, capital must be analyzed first, even though it historically appeared after landed property, since it is structurally central to bourgeois society now. “Human anatomy contains a key to the anatomy of the ape,” Marx writes,

Bourgeois economy thus supplies the key to the ancient, etc. But not at all in the manner of those economists who smudge over all historical differences and see bourgeois relations in all forms of society. One can understand tribute, tithe, etc., if one is acquainted with ground rent. But one must not identify them.^28^

“In all forms of society there is one specific kind of production which predominates over the rest,” Marx adds, “whose relations thus assign rank and influence to the others.” For instance, agriculture outranks industry before capitalism but becomes “merely a branch of industry” later. Marx exemplifies this thesis on capital’s relationship to other modes of production with ground rent, concluding that,

Ground rent cannot be understood without capital. But capital can certainly be understood without ground rent. Capital is the all-dominating economic power of bourgeois society. It must form the starting point as well as the finishing-point, and must be dealt with before landed property. After both have been examined in particular, their interrelation must be examined.

It would therefore be unfeasible and wrong to let the economic categories follow one another in the same sequence as that in which they were historically decisive. Their sequence is determined, rather, by their relation to one another in modern bourgeois society, which is precisely the opposite of that which seems to be their natural order or which corresponds to historical development.^29^

Because capital “outranks” landed property today, even though landed property appeared first historically, Marx adopts a structural-dialectical method over a simple historical narrative.^30^ Marx’s point is not that history moves unidirectionally from feudalism to capitalism, but that the dominance of capital today equips us with categories that retrospectively illuminate earlier forms. Just as human anatomy clarifies ape anatomy, capitalist relations help decode the structure of feudal ones. One cannot understand capitalism by only studying its precursors, just as one cannot fully understand humans by studying apes alone. So, Marx outlines a method that begins with developed capitalism since it clarifies earlier social forms without reducing them to it. What matters is neither the historical nor the evolutionary sequence, but the structure of the present organization of production: “For, it is only insofar as older modes of production survive within, or reappear in modified form within, capitalism that the ‘anatomy’ of the latter can provide a ‘key’ to previous social formations.”^31^

Bourgeois society overspills historical periodization. Somehow, landed property and capital combine “different times, one into another,” as Louis Althusser and Étienne Balibar helpfully put it.^32^ However, this combination is nonarbitrary since one mode of production assigns “rank and influence to the others.” To grasp this complexity, Marx sets aside history and takes up society, shifting epistemic referents from the past to the present organization of bourgeois society, a society whose rankings do not conform to historical sequence. Marx proposes to analyze how elements that preexist capitalism, such as rent, combine capitalist social relations to form a historically specific social structure. Since landed property and rent persist under capitalism, Marx must also analyze how the structure in which they are embedded reorganizes itself according to different connections and constraints. “The form of landed property with which we are dealing is a *specific* historical form,” Marx writes later, “a form *transformed* [verwandelte] by the intervention of capital and the capitalist mode of production.”^33^ Landed property extracts rents before and during capitalism. What changes is *how* this surplus is extracted.

### English Colonialism

Marx praises English political economists for solving the problem of ground rent and suggests their perspicacity stems from a particular mode of plantation colonialism,^34^

A second factor influencing the English was the knowledge they gained through their *colonies* [. . .] where, right from the start, the colonists did not seek subsistence but set up a business [. . .]. They did not act like the Teutons, who settled in Germany in order to make their home there, but like people who, driven by motives of *bourgeois production*, wanted to produce *commodities* [. . .]. That *Ricardo* and other English writers, transferred this point of view–which emanated from people who were themselves already the product of the capitalist mode of production–from the colonies to the course of world history and that they took the *capitalist mode of production* as a *prius* for agriculture in general, as it was for *their* colonists, is due to the fact that they saw in these colonies, only in a more obvious form, *without the fight against traditional relations.*^35^

Because of their involvement with plantations, English political economists could recognize therein a more “obvious form” of capitalized-agricultural production, undistorted by traditional—that is, *feudal*—social relations to land. In reflecting on English plantation colonialism, Ricardo and Anderson realized that agricultural commodities must be sold at a market price, which is below their total values. Therefore, rent derives not from occult qualities of the land, but from this excess value, which escapes exchange and is derived from labor, ultimately,

With a correct conception of rent, the first point to arise was of course that it does not originate from the land but from the *product of agriculture*, that is, from labour, from *the price of the product of labour*, for instance of wheat; from the *value* of the agricultural product, from the labour applied to the land, not from the land, and Anderson quite correctly emphasises this.^36^

Marx was still missing one crucial piece, however. According to Ricardo, rent arises from differences in agricultural labor’s productive power. The land’s “natural” qualities, such as fertility or location, are not, themselves, sources of rents, but collaterally enhance labor’s productive power by increasing output given similar capital investment on less fertile or poorly located land.^37^ According to this theory, “differential rent” depends on the land’s relative advantage as it is expressed through competition.

However, Ricardo’s thesis on agricultural labor’s differential productive power results in an embarrassing contradiction: If rent is because of differences in labor’s productive power *vis-à-vis* lands of varying quality, what explains the rent charged on the least productive land? Ricardo realized that profits from agricultural products of the worst type of land compensate for production costs only and would need to be sold *above* their values in order to generate rent–thus defying the law of value. Ultimately, Ricardo confessed that in these admittedly marginal cases, the land appears to grow value naturally, independently of labor.

### Absolute Rent

In mid-1862, Marx finally found the answer he was looking for in Rodbertus’s theory of “absolute rent.”^38^ Rodbertus persuaded Marx that agriculture and industry accumulate differently: industry employs less labor and more machinery, whereas agriculture does the opposite. Since agriculture invests in more variable capital, that is, labor, it will be less productive compared to industry, but its above-average investment in labor also means that agriculture generates more value than the social average despite inefficiency. Insofar as agriculture invests in variable capital above the social average, Rodbertus proposes that its commodities possess higher-than-average values when compared with industrial commodities. The market price of agricultural products can, and must, rise above their cost of production (*c* + *v*), thus generating a profit, yet remain below their total values (*c* + *v* + *s*), thus generating a rent. This divergence between market price and value also explains how the market prices of agricultural commodities may rise without overspilling their values and how landlords can extract rent even on land that isn’t competitive.

In sum, Rodbertus persuades Marx that different spheres of production necessitate different compositions of capital. This insight provides the basis for Marx’s theory of absolute rent: the extra payment landlords can demand, even when agricultural profit rates equal those elsewhere. Unlike differential rent, which arises from variations in productive power, and monopoly rent, which arises from exclusive ownership of space, absolute rent remains grounded in labor-power exploitation.

Marx situates his theory of rent in agriculture because of its heightened monopoly conditions and admits that “absolute rent can only be small.”^39^ However, his theory could apply to any production that invests above the social average in labor–some contemporary examples include education, healthcare, retail, communication, and creative expression. Beyond agriculture, Marx singled out resource-based extractive industries as locations of absolute rent since their costs of raw materials or constant capital are negligible. His examples include “fishing grounds, quarries, [and] natural forests.”^40^ He elaborates on the example of forestry,

Absolute rent explains certain phenomena which at first sight make rent appear to be due to a mere monopoly price. Take for instance the owner of a woodland [in] Norway [. . .]. This seems in the case of this purely natural product to be a simple monopoly surcharge. In actual fact, however, the capital here consists almost solely of variable capital laid out on labor, which therefore sets more surplus labor in motion than another capital of the same size. The value of the timber thus contains a greater excess of unpaid labor, surplus-value, than the product of capitals of higher composition.^41^

Another implication of this thesis concerns the conditions of agricultural laborers. Although agriculture has modernized in some sectors, it still relies disproportionately on variable capital, particularly in crops where mechanization is limited. This reliance makes accumulation less dependent on machinery and relative labor time and more dependent on extending work hours and absolute labor time. Critics such as Michael Heinrich and Tomba rightly caution that absolute and relative surplus value cannot be so neatly separated, since raising labor intensity is itself a source of productivity gains. Yet agriculture’s structural dependence on labor-intensive methods means that agricultural surplus is extracted primarily through coercion: lengthened working hours, heightened supervision, and strict discipline. In contemporary California, for instance, the agricultural sector depends on hundreds of thousands of precarious migrant laborers whose legal and economic vulnerability makes them especially exploitable. Here, surplus value is extracted not through constant capital but through authoritarian labor regimes, confirming that agriculture’s reliance on variable capital entrenches draconian forms of control and domination.^42^

So, rent arises not merely from increasing the laborer’s productive power–as Ricardo narrowly thought–but from the landowner’s “alien power.”^43^ In the agricultural sphere of production, capital investment and profit equalization atrophy because landlords mitigate access to land. Consequently, the industry’s organic composition of capital increases, while agriculture’s decreases. In this way, the landowner, who contributes nothing to production, continues to appropriate a portion of surplus value by erecting a limit to capitalist accumulation. With this thesis on agriculture’s above-average investment in variable capital, Marx not only explains how rents charged on noncompetitive land still conform to the labor theory of value, but also exposes how Ricardo literally assumed away the power of landowners.

Although Marx explains how landlords extract value, he also begs the question of where this class power originates, if not from within capitalism, since landlords confront capitalists as an “alien power.” From where does this alien and absolute class power originate?

### Feudalism, Really Existing

Landlords seem irreducible to, yet inextricable from, the capitalist mode of production. Surplus value seems similar to rent insofar as both presuppose private property–except that the former expresses a social relation between a productive and nonproductive class, whereas the latter unites *two* nonproductive classes, as neither landlords nor capitalists produce value. In some ways, landlords are no different from merchants, financiers, or insurers: nonproductive actors that Marx centers in *Capital*, Vol. II. But unlike those middlemen, who derive power from enhancing capital flows, landlords seem to derive power from slowing capital flows. Or perhaps rent is another instance of the capital relation, with landlords functioning as additional owners of the means of production who legally withhold them? Marx appears to be confused about this himself: Sometimes, he describes landlords as feudal holdovers; other times, he describes them as futural rent-seekers. In 1864, he describes landed property as a “superfluous nuisance” from the point of view of capital, even though “a genuine relation of production lies hidden within this irrational form.”^44^

Marx’s thesis is that landlords have been incorporated into capitalism even though they are originally alien to it. Landlords are dissimilar from the nonproductive actors of *Capital*, Vol. II, since their revenue is rent, not profit or interest. However, they cannot be so easily collapsed into the capitalist class either, since rent represents a deduction from the total social surplus. This surplus is not created by landlords but by workers who capitalists exploit. Capitalists exploit workers because of their monopoly over the means of production; correspondingly, landlords claim a portion of surplus through their monopoly of the means of *re*-production–the land. Landlords enclosed the land during an identifiably original process of expropriation, when feudalism formed the basis of capitalism. Still, landlords’ survival indicates that feudalism was not totally annihilated, as if capitalists were predestined to replace them in a trans-historical baton pass of ruling classes–a distortion that Marx himself supplies in *Communist Manifesto*. Nor does it seem as if landlords merely limped across the so-called “transition” from feudalism to capitalism since today’s landlords appear to wield even more extraordinary powers than their ancestors, monopolizing access to resources, assets, and technologies–what some have described as rentier capitalism.

Indeed, recent authors such as Cédric Durand, Yanis Varoufakis, and Jodi Dean challenge conventional narratives about capitalism’s development, arguing that the failure of communism to prevail does not necessarily signify the continuation of capitalism. Instead, they argue that capitalist relations are undergoing a systemic transformation into “neofeudalism.”^45^ According to these authors, this new system is characterized by a shift from productive capitalism to a mode of accumulation based on rents, destruction, and hoarding, driven by privilege and dependence. However, they fail to specify what relations of production give rise to this neofeudal ascendant class and how it can constitute the ruling force in this emergent mode of production. Without a clear concept of the ruling class, neofeudalism risks becoming a merely gestural framework. As Nicos Poulantzas asks, “How can we maintain that capitalism itself produces *a new class* in the course of its development?”^46^

It is no longer affordable to exaggerate the class power of capitalists and proletarians to the neglect of landlords, whose power lurks between the lines of “Original/Primitive Accumulation,” a power that tenaciously structures the capitalist mode of production even as it develops today. What is required is a more complex understanding of how the capitalist mode of production interfaces with noncapitalist modes and how such modes survive.

### Class Alliances

Pierre-Philippe Rey argues that the feudal origins of rent are neglected and proposes that the relationship between landlords and capitalists be understood as a historically specific “class alliance.”^47^ This alliance, forged during feudalism itself, was mutually beneficial but far from smooth: embryonic capitalists struck a bargain with landlords that offered the latter the capital money-form and, thus, the ability to extract rents infinitely: “Growing wealthy is an end in itself.”^48^ In return, capitalists accessed purchasable free labor, which the landlords enclosed from the soil. This class alliance, marked by cooperation and contradiction, laid the groundwork for transforming feudal relations into capitalist ones, where land and labor were increasingly commodified under capital’s normative logic of accumulation.

Marx argues that capitalists and landlords antagonize each other but that they ultimately broker an agreement “since an attack on one form of property [. . .] might cast considerable doubt on the other form.”^49^ In exchange for extracting rent, which subtracts from the social surplus, landlords allow capitalists to expropriate goods and labor once destined for peasant subsistence. For their end of the bargain, capitalists offer landlords the possibility of an infinite rent unconstrained by the cyclical or moral limits of the feudal economy. However, this very logic of accumulation inexorably erodes the vestigial authority of the landlord class. What was once an expression of ancestral juridical sovereignty becomes mere ownership, disconnected from the soil, and increasingly subordinated to the impersonal abstraction of capital. In this dialectical configuration, landlords and capitalists are mutually dependent but structurally antagonistic—bound together by a system that both sustains and transforms them.^50^

How the ruling class coalition is conceived, whether as unified or fragmented, has significant implications for identifying where political pressure can be applied and where contradictions may surface between classes, as Marx himself observed in his *Critique of the Gotha Program*.^51^ Failing to account for the complexities within class composition, such as contradictions between the proletariat and the peasantry, risks allowing reactionary forces to exploit these very divisions, perhaps by justifying violence “in the name of the people” who have been neglected. Similarly, overlooking the nuances within the ruling class coalition, particularly the structural antagonism between landlords and capitalists, risks missing critical sites of vulnerability where pressure can be applied to break apart alliances. Failing to recognize contradictions within the ruling class (and the oppressed) could lead to a fragmented struggle, where revolutionary efforts are co-opted or diverted, preventing effective organizing and coalition-building.

Marx’s concept of ground rent offers an innovative perspective on how rent, initially a precapitalist social relation, becomes integrated into the capitalist mode. In the final analysis, the capitalist mode integrates ground rent as a capitalized appropriation of surplus value, generating what Marx calls an “embryonic new mode of production.”^52^ Capital encountered alien, feudal relations such as rent and capitalized them with collateral side effects: The ancestral right to the land was turned into a real estate market and rents generated from the sale and ownership of real estate ultimately atrophied surplus value. In this analysis, Marx applied his most empirical and scientific methods, solving an old problem with a groundbreaking insight and finding the scoop to a scandal that was right under everyone’s noses. The scoop was that Ricardo and other political economists twisted themselves into knots to try to explain away the truth that was out in the open and obvious to everyone who wasn’t a paid spokesperson for capitalism: landlords charge rent not because they *provide* housing; they charge rent because they *withhold* it.

## Speculative Expropriation

Broadcast ownership (i.e., ownership scattered broadly among settlers rather than concentrated in a few estates) and an ostensible abundance of land posed fundamental challenges to the capitalist social relations discussed in the previous sections. While Europeans invaded the New World with force and fiscal ambition, they quickly discovered that it posed new problems their peculiar relation of production could not overcome: the persistent sovereignty of Indigenous peoples and the unsettling plenitude of the land itself. As Marx notes in the last chapter of *Capital*, Vol. I, land challenges capitalist social relations since those relations presuppose scarcity and vulnerability.^53^ Unmoored from the constraints of private property, settlers might “go native,” live off the land, or abandon work altogether. In this context, capitalism did not begin by fencing people out—first, it had to fence people in.

### Systematic Colonization

Marx’s engagement with Edward Gibbon Wakefield extends beyond the chapters on original accumulation; in fact, Wakefield appears explicitly and throughout Marx’s analysis of ground rent. Engels retains some of Marx’s references to Wakefield in *Capital*, Vol. III, but omits others. These omissions undermine the centrality of Wakefield’s ideas to Marx’s critique of capitalist social relations to land and rent. For example, in a paragraph from his draft manuscripts that Engels edited for *Capital*, Marx explains how pricing works for “things which have no *value* in and for themselves,” interrupting the clause with a footnote Engels deleted: “See Wakefield.” “For a thing to be sold,” Marx concludes, in reference to Wakefield, “it simply has to be capable of being monopolised and alienated.”^54^ In *Capital*, Engels moves this paragraph later in Marx’s presentation for added emphasis. However, deleting Marx’s reference to Wakefield here de-emphasizes how trans-Atlantic capitalist property relations necessitated artificial pricing mechanisms in order to induce wage dependency.^55^ So, unlike original accumulation, where colonialism functions as an example of capitalist expropriation, Marx uses colonialism here as a counterexample, insofar as colonial, broadcast ownership prevents vulnerability and dependency necessary for ground rent. The West is both confirmation and contradiction.

Wakefield’s “systematic colonization” was designed to resolve the British Empire’s crises of overpopulation and surplus capital. He proposed using colonies as both a safety valve for excess labor and a market for overripe capital. By setting an artificial price on land, the state could force settlers to work before becoming proprietors, using land-sale revenues to import more of Britain’s underclass. This reproduced in the West what the Enclosures had done in Britain, ensuring colonial labor’s subordination to colonial capital. As Onur Ulas Ince notes, this “hydraulic circuit” channeled surplus labor and capital abroad while sustaining prosperity in the metropole.^56^

Ultimately, systematic colonization failed. In *Capital*, Vol. I, Marx observed that Wakefield’s scheme would be rendered obsolete by the rise of speculative joint-stock companies, which reshaped land relations in ways Wakefield could not have anticipated. Initially, speculative land markets appeared to benefit colonization by driving land sales, but they soon interfered with the process they were meant to facilitate since speculators had no interest in settling or farming the land themselves; they were concerned only with making a profit through land-related financial speculation.

In a paragraph missing from the recent 2024 English translation of *Capital*, Vol. I, Marx explains that the US Civil War generated an enormous public debt owed to a “finance aristocracy of the vilest type.” In *Le Capital*, Marx explains that the United States, still reeling from the war, could not repay this debt, so it offered “immense tracts of public land to speculative companies” instead.

This “very rapid centralization of capital” permitted speculators, rather than farmers, to control the West.^57^ Speculators, driven by the desire for money for money’s sake, quickly and irreparably reshaped the entire landscape of colonization: “The advance of capitalist production and an accompanying increase in pressure from the government have made Wakefield’s prescription superfluous.”^58^ Thus, the settler state became a real estate scam *disguised* as a country.

### Speculative Imperialism

Marx’s views on speculation develop throughout his work, reflecting his increasing attention to the role of finance in the expansion of capitalist relations. In his *Tribune* article, “The Economic Crisis in Europe,” published on October 9, 1856, Marx remarked on the “universality of the rage” for speculation that had swept through Europe–which was especially prescient since the Panic of 1857 was mere months away.^59^ By the 1860s, Marx was increasingly insistent on how speculation and finance had become central to spreading capital beyond Britain.^60^

Marx saw speculation as a fundamental departure from earlier colonial models. The “British Empire”–a term coined in 1579 when ambition exceeded achievement–did not expand westward through wage labor or industry but through speculative companies. By the seventeenth century, England had failed to secure lasting colonies in the Americas, subdue Ireland, or dominate the Caribbean slave trade. These failures framed the founding of the East India Company in 1600, whose publicly traded shares marked both a pivot in imperial strategy and a milestone in financial speculation. Unlike aristocratic pursuits of honor, speculative ventures were merchant-driven and profit-oriented, and were soon emulated by the Dutch East India Company. As James Shapiro puts it: “The death of chivalry coincided with the birth of empire.”^61^

Speculation inflated company stocks, spurred buying frenzies, and self-destructed into a series of financial catastrophes, such as the infamous South Sea Bubble. These financial disasters compelled Parliament to pass the *Bubble Act* of 1720, which, *inter alia*, required colonially speculative enterprises to acquire a state-sanctioned Royal Charter. Soon, royally chartered companies, such as the Hudson’s Bay Company, which was only recently bankrupted in 2025, were granted charters and played central roles in organizing and administering lands and territories that had yet to be integrated. In this way, capital did not take root in the ‘economic base’ of colonial society, but instead emerged within its ‘social superstructure’: joint-stock companies, empowered by the state, effectively *hijacked* non-capitalist direct producers, subsuming their labor and resources within a world market. By “hijack,” I do not mean that capital simply “steals,” but that joint-stock companies forcibly redirected non-capitalist producers and their outputs into circuits of abstract wealth, transforming subsistence activity into commodified labor and resources for the world market.^62^ Thus, imperial expansion became a means of embedding capitalist relations in territories that had yet to be fully recruited into global capitalism, speculatively infiltrating non-capitalist superstructures.^63^

Settlers actively engaged in speculative, unstable, and dynamic land bubbles, too. Unlike Europe, where land was held under multiple overlapping lordships and often lacked secure, unchallengeable chains of title, New England bypassed feudal landholding systems (including their colonial mutations: New France’s *seigneurial* system and New Spain’s *haciendas*) by embedding the legal constitution of the mortgage. The term “mortgage” derives from the medieval French phrase meaning “death pledge,” referring to the pledge “dying” either when the debt is fully repaid or when the land is foreclosed. Secured against the borrower’s property, a mortgage granted lenders the right to repossess and sell the land should the borrower default. As settlers pushed westward, mortgages expanded alongside them. These loans commonly spanned five years but often required steep deposits of up to 50 percent. Predictably, many borrowers failed to meet their obligations, leading to foreclosures that returned land to the state, which then sold or granted it to large companies—perpetuating a cycle of speculation, distribution, and recentralization. This continual buying, selling, and reselling created new chains of title and transformed land into an alienable and fungible commodity. Ultimately, the mortgage was one of the essential instruments that turned the West’s ostensible abundance of land into a speculative market, reshaping its economic and social future.

However, the speculators could not invent land out of thin air, especially if it had already been claimed. Which it was.

Both Indigenous peoples and British imperialists laid competing claims to the land. But, tensions escalated when settlers accused the Crown of ceding “their” land to Indigenous peoples, fomenting a class war between imperial royalists and colonial elites whom CLR James famously called “Big Whites.”^64^ In a surprising twist, American-settler-colonial “Big Whites” began *defending* Indigenous rights, but not out of genuine solidarity. No, the Big Whites argued that protective British legislation, especially the *Royal Proclamation* (1763), unjustly deprived Indigenous peoples of the liberty to sell their lands to settlers freely.^65^

To circumvent such protections, speculators introduced the practice of preemptive land sales. Novel to the New World, preemption did not grant full ownership but only the nominal right of first refusal to purchase Indigenous land at a federally set price. In practice, this right functioned as a credit instrument: it allowed settlers to leverage future purchases against present claims, turning land into a financial asset before it was alienated.^66^ This mechanism enabled settlers to sidestep direct confrontations over Indigenous and imperial claims by installing capitalist property relations in *advance* of dispossession. Preemptive sales thus exemplify my interpretation of Marx’s *Grundrisse*, in which I locate a theory of nonlinear capitalist property relations. Such relations, exemplified here by pre-emptive land sales, are non-linear, as they project forward, conditioning and anticipating the physical expropriation of existing occupants.

### Colonization, Inc

The “Canadian” West was sold before it was settled. In 1881, Prime Minister John A. Macdonald–a Canadian “Big White”–expanded the *Dominion Lands Act* to include “colonization companies,” granting one 10 million acres of land to offset the $25 million subsidy promised to the Canadian Pacific Railway. These companies, often led by Conservative Party insiders, were not simply agents of immigration but instruments of speculative expropriation. Like their predecessors in the British Empire and the US, they purchased land at discounted rates and sold it to settlers for profit, often bundling transportation, tools, and seed into package deals designed to inflate land value through artificial concentration. But beneath the promotional rhetoric of cultivation and progress lay a financial logic that converted land into a speculative commodity, a commodity whose value increased not through use, but abstraction. In the settler colony, capitalist property relations were not the outcome of productive labor but its precondition.

Nowhere is this clearer than in the case of the Métis at South Branch. Their lands were sold to colonization companies like the Prince Albert Colonization Company—controlled by local Tory elites—without their consent. Despite accepting similar petitions from Anglo-Métis and settler communities at North Branch, the Canadian government systematically denied land title to the French-speaking Métis, who increasingly suspected linguistic and religious discrimination. Métis land claims were rejected not because of procedural irregularity but because the territory had already been granted to corporate interests that were more concerned with urban speculation than agricultural settlement. When residents attempted to renew their deeds, they discovered the entire parish had been deeded to the colonization company.^67,68^

Preemptive land sales, colonization companies, and mortgages installed capitalist property relations *before* actual dispossession. This speculative sequencing—claiming land, selling rights, and generating title before removing Indigenous occupants—demonstrates that capitalist expansion does not unfold through a linear evolution of forms (a point Tomba agrees with); it also emerges through violent and financial impositions that *cause* the very conditions they presuppose.

Speculators realized land was not an inherently fungible asset but necessitated substantial investment and a legal framework to transform it into an alienable commodity. To achieve this, they revived and invented legal fictions first developed in England—most notably the concept of “fee-simple” ownership and the mortgage, which emerged as a distinctive feature of colonization in New England. Unlike in Europe, where landholding was often layered with customary or feudal obligations, fee-simple in the New World stripped land down to a unit of absolute, alienable ownership, an innovation that was essential to both capitalism and settler colonialism. Mortgages were instrumental also because they allowed creditors to tie debt obligations to specific plots of land, effectively excluding competing claims that had hindered settlement efforts in New France and New Spain. Speculation increased land sales, generated fee-simple title chains, legitimized land seizures from Indigenous peoples, and thus, facilitated population replacement.

Speculative expropriation involved quantifying land through systematic colonization and its qualitative transformation into a commodity, facilitated by fictitious capital. Through these processes, Indigenous peoples were dispossessed and their territories sold to joint-stock companies and individual homesteaders, who actively contested Indigenous claims, thereby consolidating settler colonial control and population replacement, ominous features that continue to structure settler colonialism today—most visibly in the juridical and financial fictions that sustain Israel’s settlement of Palestinian land.^69^

## Conclusion

Settler colonialism seems to defy Marx’s analyses of original accumulation and ground rent. In original accumulation, Marx argues that “expropriating the direct agricultural producer is the basis of the whole process.” In ground rent, he assumes “landlords will never let *gratis*.” In the New World, however, emplacing agricultural producers and letting the land (nearly) for free seemed to have been “the basis for the whole process,” instead. Unlike landlords, who took the land away, speculative expropriators gave it away, often before extinguishing Indigenous possession, as evidenced by preemptive fee-simple land sales. Speculative expropriators placed direct producers on the land, then threatened to repossess it unless conditions were met and mortgage debts were honored. Of course, land was not given away to anyone. Eligible proprietors satisfied certain social conditions related to gender and especially race, conditions that indebted new land owners to the state, as well as a new mode of social difference epitomized by frontier white cultivator masculinity. In a racially stratified social context made possible because of Indigenous genocide, debt and credit proved to be sufficient for controlling settler proprietors. Western expropriation balanced a contradiction: it introduced private property in land while ensuring that those who previously lived on it couldn’t stay unless they conformed.

I have been arguing that “primitive” or “original” accumulation is an oxymoron since capitalism’s threshold is irreducible to the normative logic of accumulation. After contextually shifting to original expropriation, I reconstructed Marx’s analysis of ground rent from his manuscripts and notebooks, and then reanimated this synthesis as a framework for understanding how capitalism interfaces with a noncapitalist mode (i.e., feudalism). Finally, I suggested that a battery of colonial and financial constructs–mortgages, preemptive land sales, and speculative fee-simple real estate markets–emplaced settlers, invested them in Indigenous dispossession, and then expanded capitalism globally. Throughout, I followed Marx west, where I located a typology of original accumulations and offered my own original theorization: *speculative expropriation*.

A key advantage of *speculative expropriation* is its portability. My concept can be used to analyze forms of enclosure not only in early settler colonies, but across imperial and postcolonial contexts. For instance, seventeenth-century Iberian colonial ventures reinvested in slave-trade speculation, effectively combining human and territorial expropriations into speculative capital circuits.^70^ More recently, Puerto Rico’s debt regime and land-title contests reveal a modern speculatory logic: investors leverage usurious debt instruments to dispossess land and restructure property relations.^71^ Haiti’s massive “independence debt” to France (1825) and subsequent fiscal obligations likewise illustrate how speculative expropriation operates through sovereign debt rather than direct land seizure.^72^ And, as has already been discussed, in Palestine, Israeli settlement policies similarly deploy legal and financial fictions to convert occupied land into alienable property, masking dispossession and genocide beneath a façade of legality.^73^ Finally, the 2008 financial crisis demonstrated how mortgages and derivatives became instruments of dispossession, transferring millions of homes from working-class families to financial institutions under the guise of market rationality.^74^ Together, these cases show how speculative expropriation offers a framework for analyzing the continuities of colonial and capitalist domination across contexts.

This study has one critical limitation that necessitates future research, however. In their analyses, Marx and Rey both assume that the land has no social relation independent of or before original expropriation.^75^ Although Marx and many of his readers criticize expropriators with trenchant and damning criticisms, they also seem to traffic a quantitative, and perhaps “economistic,” understanding of the land and what it represents for non-Europeans.

For capitalism to take root in the West, it needed to emplace an exploitative, accumulative, and proprietorial social relation. Yet, as in Europe, the capital relation did not take root *terra nullius*: it first “discovered” and then hijacked an extant, *non*capitalist social relation. Even capitalism could not annihilate this relation, though. Today, Indigenous social relations to land are vibrant and defiant in our communities, and militant demand for “Land Back.” This alternative eco-political imaginary opens up for us only when we seriously consider Indigenous social relations to land that preexist, and survive alongside, the capitalist relation. It is necessary to conceive capitalism in more intersectional terms that not only attend to its quantitative thrust and geographic spread but also the *qualitative* shift it represents in social relations to land. My study discussed social relations to land from Europe only. The remaining work must consider social relations from an unapologetically Indigenous perspective of “grounded normativity.”^76^

What is at stake here is whether social relations to land are marginal or central to the capitalist mode of production and whether struggles over land, housing, and space are immanent sites of class war. If we ossify original/primitive accumulation as a historical schema that inexorably ends with the proletariat, then our theory of class war will necessarily treat decolonial and national struggles as auxiliary at best or counterrevolutionary at worst. If we refuse to consider how precapitalism persists and even develops differently under capitalist relations of production, we will miss practical and concrete alternatives to private property. Unless we conceive expropriation in more capacious terms that overspill binaries and schemas, we will undertheorize expropriation, fail to recognize partitioned modes of capitalist production, and thus, misdiagnose solutions for transcending. We cannot afford an obscure or indeterminate theory of expropriation amid today’s struggles over land, housing, and the unequal and racialized distribution of space–from *favelas* in Brazil to Indian reservations in Saskatchewan and olive groves in the state of Palestine.

## Notes

1. Karl Marx, *Capital: Critique of Political Economy*, ed. Paul North, trans. Paul Reitter, with Wendy Brown and William Clare Roberts (Princeton University Press, 2024 [1867]), 701.

2. Karl Marx, *Marx’s Economic Manuscript of 1864-1865*, ed. Fred Moseley, trans. Ben Fowkes (Koninklijke Brill NV, Leiden, The Netherlands, 2016 [1864-65]), 749.

3. Marx, *Marx’s Economic Manuscript of 1864-1865*, 715–16.

4. Ibid.

5. Marx’s original manuscript was first published in German in 1992 and translated into English in 2015, so only recently have readers been able to compare Marx’s draft with Engels’s version. Although Marx once claimed that, “Whatever shortcomings they may have, the advantage of my writing is that they are an artistic whole,” it still seems that many holes remain on questions of land, colonialism, and how exactly that capitalism came to dominate the West despite lacking its “permanent foundation”—that is, feudal-European land tenure. Karl Marx and Friedrich Engels, *Collected Works. Vol. 42: Marx and Engels: 1864-68* (Progress Publ. 1987), 173.

6. Karl Marx, *Karl Marx, Frederick Engels Collected Works: Marx and Engels: 1845-1847*, with Frederick Engels (International Publishers, 1976), 5:84.

7. Marx, *Capital*, 537.

8. Despite his earlier warning to Engels that “You can’t compress a course of political economy into one hour!” Karl Marx, “Letters: Marx-Engels Correspondence 1865,” May 20, 1865, https://www.marxists.org/archive/marx/works/1865/letters/65_05_20-abs.htm.

9. Karl Marx, “Economic Manuscripts: Value, Price and Profit,” 1865, 1969, https://www.marxists.org/archive/marx/works/1865/value-price-profit/.

10. Later, in the 1880s, Marx will say so explicitly to Vera Zasulich. Teodor Shanin, *Late Marx and the Russian Road: Marx and the Peripheries of Capitalism*, Monthly Review Press Classic Titles Ser, v. 26 (Monthly Review Press, 1983).

11. David Graeber and D. Wengrow, *The Dawn of Everything: A New History of Humanity* (Signal, 2021).

12. David Harvey, *The New Imperialism* (Oxford University Press, 2005).

13. Massimiliano Tomba, *Marx’s Temporalities*, Historical Materialism Book Series, vol. 44 (Brill, 2013); Massimiliano Tomba, “Accumulation and Time: Marx’s Historiography from the Grundrisse to Capital,” *Capital & Class* 37, no. 3 (2013): 355–72.

14. Kevin Anderson, *The Late Marx’s Revolutionary Roads: Colonialism, Gender, and Indigenous Communism* (Verso, 2025); Amy Allen, “Dripping with Blood and Dirt from Head to Toe: Marx’s Genealogy of Capitalism in Capital , Volume 1,” *The Monist*, 105, no. 4 (2022): 470–86; Amy Allen, “Universality, Necessity, and Progress: Marx and the Problem of History,” *Emancipations: A Journal of Critical Social Analysis*, 3, no. 3 (2024); Jason Read, “Primitive Accumulation: The Aleatory Foundation of Capitalism,” *Rethinking Marxism*, 14, no. 2 (2002): 24–49.

15. Amy Allen, “Dripping with Blood and Dirt from Head to Toe: Marx’s Genealogy of Capitalism in Capital , Volume 1,” *The Monist*, 105, no. 4 (2022): 470–86; Amy Allen, “Universality, Necessity, and Progress: Marx and the Problem of History,” *Emancipations: A Journal of Critical Social Analysis*, 3, no. 3 (2024).

16. Kevin Anderson, *The Late Marx’s Revolutionary Roads: Colonialism, Gender, and Indigenous Communism* (Verso, 2025), 149.

17. Karl Marx, *Das Kapital. Kritik Der Politischen Ökonomie. Von Karl Marx. Erster Band. Buch I: Der Produktionsprocess Des Kapitals.*, 1st ed. (Otto Meissner, L.W. Schmidt, 1867), 701, https://oll.libertyfund.org/titles/das-kapital-kritik-der-politischen-oekonomie-buch-1-1867.

18. There is no English translation of the first German edition of *Capital*, Vol. I. Samuel Moore and Edward Aveling’s seminal English translation was based on the third German edition. I translate Marx’s original German as: “The expropriation of the workers from the soil forms the foundation of the whole process. We must therefore consider it first. Its history unfolds in different phases and in a different sequence. Only in England, which we therefore take as our example, does it possess its classical form.”

19. Karl Marx, *Marx Engels Werke (MEW)* (Berlin, 1983), 23:744.

20. William Clare Roberts, “The French Reconstruction of Capital, 1872-1875,” in *Capital, Vol. I* (Princeton University Press, 2024).

21. Marx, *Capital*, 690.

22. Karl Marx, *Le Capital*, 1st ed., trans. Joseph Roy (Maurice Lachatre, 1875), 341, https://archive.org/details/ldpd_13051551_000/page/341/mode/2up.

23. Peter Hudis, “Pathways to Social Development: Rosa Luxemburg’s Studies on the Anthropology and Sociology of Imperialism,” *Inter-Asia Cultural Studies*, 24, no. 1 (2023): 129–42.

24. Karl Marx and Friedrich Engels, *Collected Works. Vol. 31: Marx and Engels: 1861-63*, Collected Works 31 (Lawrence & Wishart, 1989), 593, note 99.

25. Marx, *Marx’s Economic Manuscript of 1864-1865*, 719.

26. Enrique Dussel and Karl Marx, *Towards an Unknown Marx: A Commentary on the Manuscripts of 1861-63*, ed. Fred Moseley, trans. Yolanda Angulo, Routledge Studies in the History of Economics (Routledge, 2002), xx; Susumu Takenaga, “Marx on Rent: New Insights from the New MEGA,” *The European Journal of the History of Economic Thought*, 25, no. 5 (2018): 926.

27. Dussel and Marx, *Towards an Unknown Marx*, 83.

28. Karl Marx, *Grundrisse*, trans. Martin Nicolaus, Penguin (Penguin, 1993 [1857]), 105.

29. Marx, *Grundrisse*, 106–7.

30. Which is perhaps another reason why he concluded *Capital*, Vol. I, with an analysis of capital’s beginning.

31. Stuart Hall, “Marx’s Notes on Method: A ‘Reading’ of the ‘1857 Introduction’ [1974],” in *Selected Writings on Marxism*, Stuart Hall: Selected Writings (Duke University Press, 2021), 44.

32. Louis Althusser and Étienne Balibar, *Reading Capital: The Complete Edition*, trans. David Fernbach and Ben Brewster (Verso, 2015), 330.

33. Marx, *Marx’s Economic Manuscript of 1864-1865*, 713.

34. Continental political economists, like the Physiocrats, mistakenly believed that land generates value but correctly understood that commodities are sold at their values; by contrast, the mercantilists thought profits were due to selling dearly, but, like the Physiocrats, they overlooked the connection between labor and value: both camps responded to the same problem unsuccessfully.

35. Marx and Engels, *MECW 31* [1861–63], 459–60.

36. Marx and Engels, *MECW 31* [1861–63], 372.

37. For example, let’s assume two capitalists produce the same commodity but lease land from different landlords. The first capitalist routinely purchases coal to fuel a steam-powered turbine necessary for production, but the second harnesses a natural river, which powers his water turbine for free. Consequently, the first capitalist, who buys fuel, produces *at* the social average, whereas the second capitalist, who uses the river, produces *below* the average. Eventually, the capitalist who produces below the average will accumulate a surplus profit as the river is an exclusive production condition. Therefore, the river is *not* a “natural” source of value but a collateral power-up. The landlord draws rents *not* from values “inherent” to land, but from the difference in individual versus social production: “rent is always the difference between the produce obtained by the employment of two equal quantities of capital and labor.” D. Ricardo, *On the Principles of Political Economy, and Taxation*, Everyman’s Library (John Murray, 1821), 59.

38. As I mentioned previously, Marx was attempting to finish the third chapter of his 1859 *Contribution* when he discovered Rodbertus. At the time, Marx had just written five notebooks, which later became Parts 2–4 of *Capital*, Vol. I. He planned to analyze machinery and relative surplus value next–but, instead of continuing with this investigation, Marx devoured Rodbertus and then used him as a platform from which to dive into a critique of the history of ideas about surplus value. Marx would not return to his original plan to analyze relative surplus value until he filled another twelve notebooks in January 1863.

39. Marx, *Marx’s Economic Manuscript of 1864-1865*, 759.

40. Ibid., 759.

41. Ibid., 756.

42. Alvaro Medel-Herrero et al., “Documenting Working Experiences of Agricultural Workers in California,” *The International Journal of Sociology of Agriculture and Food*, 29, no. 2 (2023): 15–34, document the working and living conditions of Latinx agricultural workers in California—around 800,000 employed annually, about half undocumented. Workers characterize their conditions as “little better than slave labor,” citing systematic abuse, exploitation, discrimination, and institutional marginalization. Additionally, studies of H-2A visa labor regimes show that agricultural workers with precarious status suffer more labor violations, owing in part to the limited capacity of inspection agencies and the structural power imbalance in employer-tied contracts. Arezoo Jafari et al., “Enhancing Detection of Labor Violations in the Agricultural Sector: A Multilevel Generalized Linear Regression Model of H-2A Violation Counts,” arXiv:2306.04003, preprint, arXiv, June 6, 2023, https://doi.org/10.48550/arXiv.2306.04003.

43. Marx, *Marx’s Economic Manuscript of 1864-1865*, 749.

44. Ibid., 721.

45. Jodi Dean, *Capital’s Grave: Neofeudalism and the New Class Struggle* (Verso, 2025); Yanis Varoufakis, *Technofeudalism: What Killed Capitalism* (Melville House, 2024); Cédric Durand, *How Silicon Valley Unleashed Techno-Feudalism: The Making of the Digital Economy*, trans. David Broder (Verso, 2024).

46. Nicos Poulantzas, “On Social Classes,” *New Left Review*, no. I/78 (April 1973): 38.

47. Pierre-Philippe Rey, *Class Alliances*, trans. James F. Becker, vol. 12, International Journal of Sociology 2 (Taylor & Francis Ltd., 1982).

48. Marx, *Grundrisse*, 270.

49. Marx and Engels, *MECW 31*, 278.

50. As Marx explains in his *Manuscripts of 1864-1865*, “It is one of the great results of the capitalist mode of production [that] on the one hand it completely detaches landed property [*völlig loslöst*] from relations of lordship and servitude, while on the other hand it completely separates the land [*gänzlich trennt*] as a *condition of labour* from landed property and the landowner [. . .]. It undoes the connection to such an extent that the landed proprietor can spend his entire life in Constantinople, while *his own* landed property remains in Scotland. Landed property thus receives its *purely economic form* by the stripping away of all its former political and social embellishments and admixtures that are denounced as uselessly and absurdly superfluous by the industrial capitalists themselves, and by their theoretical spokesmen”. Marx, *Marx’s Economic Manuscript of 1864-1865*, 716–17.

51. There, Marx critiques the Gotha Program's failure to address the abolition of landlordism in the transition to socialism. He argues that landlords, like capitalists, are part of the ruling class that exploits workers, and that their control over land must be dismantled for a true socialist transformation. For Marx, ending private property in land is essential to eliminate ground rent and ensure that resources are collectively owned and controlled by the working class, not by parasitic landlords.

52. Karl Marx, *Capital, Vol. 3* (Penguin, 1993), 814–15.

53. “The capitalist mode of production is based on the land being expropriated from the majority of the people. In a free colony, in contrast, the majority of the land still belongs to the people, and thus anyone who settles on it can turn it into his private property and also his individual means of production.” Marx, *Capital*, 696.

54. Marx, *Marx’s Economic Manuscript of 1864-1865*, 727; Marx, *Capital, Vol. 3*, 772.

55. Engels omits another passage where Marx discusses Wakefield in the context of absolute rent. In this excised paragraph, Marx explains that the problem of absolute rent paid on marginal land is not resolved by turning to the colonies, Even in colonies *grants* of big uncultivated estates to particular individuals as their private property constitute a burdensome restriction on colonisation (since they restrict the field of employment) and if this were done on a large scale it would destroy the colony’s character as a colony, and it would still do nothing to establish the conditions enjoyed by countries of ancient civilisation there, as Wakefield has correctly remarked. This point did not prevent the same Wakefield from advocating the establishment of an artificial land price in the colonies. Marx, *Marx’s Economic Manuscript of 1864-1865*, 744–45.

56. Onur Ulas Ince, *Colonial Capitalism and the Dilemmas of Liberalism* (Oxford University Press, 2018), 115.

57. Marx, *Le Capital*, 347.

58. Marx, *Capital*, 701.

59. Karl Marx, “The Economic Crisis in Europe by Karl Marx 1856,” September 26, 1856, https://www.marxists.org/archive/marx/works/1856/10/09.htm.

60. For example, in his *Manuscripts of 1864-65*, he draws attention to the speculative practices of groups like *La Bande Noire*, a speculative real estate group active during post-revolutionary France that flipped castles, buying them for next to nothing after the National Constituent Assembly expropriated them from nobles, and then demolishing and salvaging their building materials. This example highlights how speculation could reshape land ownership and value through the manipulation of its material use, rather than its agricultural productivity or social utility. Marx, *Marx’s Economic Manuscript of 1864-1865*, 796, note 77. Moreover, in his analysis of differential rent, Marx suggests that speculative real estate markets generate rent conditions by casting a “reflection of the capital investment in the cultivated lands.” Marx, *Capital, Vol. 3*, 826. Finally, in a letter to Nikolai Danielson in 1879, Marx wrote that the immense joint-stock companies associated with railways “embraced the whole world in a network of financial swindling and mutual indebtedness” Karl Marx, “Letters: Marx-Engels Correspondence 1879,” accessed November 6, 2023, https://www.marxists.org/archive/marx/works/1879/letters/79_04_10.htm.. Railway systems allowed previously isolated capitalist centers to expand their reach and integrate new markets.

61. James S. Shapiro, *1599 - a Year in the Life of William Shakespeare* (Faber and Faber, 2005), 274.

62. Althusser frequently uses the French verb “*détourner*” in *Reading Capital* to describe how Marx “diverts” or “redirects” the concepts of classical political economy into a scientific critique. While the standard English renderings tend toward “divert” or “redirect,” my use of *hijack* is intended to capture the forceful re-appropriation implied by *détournement* in the Althusserian lineage Althusser and Balibar, *Reading Capital*.

63. As Jairus Banaji notes, under commercial capitalism, “the subjection of labor to capital occurred in a form that did not presuppose its simultaneous subordination through the labor process.” Jairus Banaji, *A Brief History of Commercial Capitalism* (Haymarket Books, 2020), 86. Speculative companies exemplified this logic by subordinating noncapitalist producers without directly reorganizing their production, instead channeling their labor and resources into circuits of accumulation.

64. C. L. R. James, *The Black Jacobins: Toussaint l’Ouverture and the San Domingo Revolution*, 2nd ed., rev. (Vintage Books, 1989).

65. As Robert Williams laments, “even the most hardened land-market capitalist assumed the mantle of zealous advocate of the Indians’ natural-law right to engage in unregulated real-estate transactions” (cited in Greer 2017, 384).

66. While not formally a credit contract, preemption functioned in quasi-credit terms by binding future purchase to present claims, effectively financializing land before dispossession. This anticipatory logic links pre-emption to mortgages and debt more broadly, both of which tethered settlers to state-backed financial infrastructures.

67. Jean Teillet, *The North-West Is Our Mother: The Story of Louis Riel’s People, the Métis Nation*, First edition (Patrick Crean Editions, an imprint of HarperCollins Publishers Ltd, 2019), 316.

68. Canada’s speculative expropriation underscores Karl Polyani and Allan Greer’s point that “‘land’ per se is not inherently a fungible asset.” Greer, *Property and Dispossession*, 410; Karl Polanyi et al., *The Great Transformation: The Political and Economic Origins of Our Time*, 2. Beacon paperback ed. [reprinted] (Beacon Press, 2010). “The trick was to pretend that territory was already part of the settler property regime, and by that fiction make it so.” Greer, *Property and Dispossession*, 221–22.

69. Lorenzo Veracini, *The Settler Colonial Present* (Palgrave Macmillan, 2015); Noura Erakat, *Justice for Some: Law and the Question of Palestine* (Stanford University Press, 2019).

70. John Huxtable Elliott, *Empires of the Atlantic World: Britain and Spain in America, 1492 - 1830*, 1 (Yale University. Press, 2007).

71. Rocío Zambrana, *Colonial Debts: The Case of Puerto Rico*, Radical Américas (Duke University Press, 2021).

72. Kim Oosterlinck et al., “The Odious Haitian Independence Debt,” *Journal of Globalization and Development* (Berlin), 13, no. 2 (2022): 339–78.

73. Erakat, *Justice for Some*.

74. David Harvey, *The Enigma of Capital: And the Crises of Capitalism* (Oxford University Press, 2011); Manuel B. Aalbers and Anne Haila, “A Conversation about Land Rent, Financialisation and Housing,” *Urban Studies*, 55, no. 8 (2018): 1821–35.

75. “One never finds behind the object ‘land’ a symmetrical and positive social relation,” writes Rey Rey, *Class Alliances*, 12:27.. Marx is also quite clear: “The value of land is nothing but the price which is paid for capitalized rent” Karl Marx and Friedrich Engels, *Collected Works. Vol 32: Karl Marx: 1861 - 63* (International Publishers, 1989), 299.

76. Glen Sean Coulthard, *Red Skin, White Masks: Rejecting the Colonial Politics of Recognition*, Indigenous Americas (University of Minnesota Press, 2014).

